# Melatonin Inhibits Endoplasmic Reticulum Stress and Epithelial-Mesenchymal Transition during Bleomycin-Induced Pulmonary Fibrosis in Mice

**DOI:** 10.1371/journal.pone.0097266

**Published:** 2014-05-12

**Authors:** Hui Zhao, Qing-Qing Wu, Lin-Feng Cao, Hou-Ying Qing, Cheng Zhang, Yuan-Hua Chen, Hua Wang, Rong-Ru Liu, De-Xiang Xu

**Affiliations:** 1 Department of Toxicology, Anhui Medical University, Hefei, China; 2 First Affiliated Hospital, Anhui Medical University, Hefei, China; 3 Second Affiliated Hospital, Anhui Medical University, Hefei, China; French National Centre for Scientific Research, France

## Abstract

Several reports indicate that melatonin alleviates bleomycin (BLM)-induced pulmonary fibrosis in rodent animals. Nevertheless, the exact mechanism remains obscure. The present study investigated the effects of melatonin on endoplasmic reticulum (ER) stress and epithelial-mesenchymal transition (EMT) during BLM-induced lung fibrosis. For the induction of pulmonary fibrosis, mice were intratracheally injected with a single dose of BLM (5.0 mg/kg). Some mice were intraperitoneally injected with melatonin (5 mg/kg) daily for a period of 3 wk. Twenty-one days after BLM injection, lung fibrosis was evaluated. As expected, melatonin significantly alleviated BLM-induced pulmonary fibrosis, as evidenced by Sirius red staining. Moreover, melatonin significantly attenuated BLM-induced EMT to myofibroblasts, as determined by its repression of α-SMA expression. Further analysis showed that melatonin markedly attenuated BLM-induced GRP78 up-regulation and elevation of the cleaved ATF6 in the lungs. Moreover, melatonin obviously attenuated BLM-induced activation of pulmonary eIF2α, a downstream target of the PERK pathway. Finally, melatonin repressed BLM-induced pulmonary IRE1α phosphorylation. Correspondingly, melatonin inhibited BLM-induced activation of XBP-1 and JNK, two downstream targets of the IRE1 pathway. Taken together, these results suggest that melatonin alleviates ER stress and ER stress-mediated EMT in the process of BLM-induced pulmonary fibrosis.

## Introduction

Idiopathic pulmonary fibrosis, characterized by fibroblast proliferation and extracellular matrix remodeling, is a chronic pulmonary disease of unknown origin ultimately leading to death [1], [2]. Bleomycin (BLM), a drug widely used as an antineoplastic, causes a dose-dependent interstitial pulmonary fibrosis [3]. Intratracheal instillation of BLM into the lungs of rodent animals causes alveolar cell damage, an inflammatory response, epithelial-mesenchymal transition (EMT), fibroblast proliferation and subsequent extracellular matrix deposition, resembling human fibrotic lung disease [4]. BLM-induced pulmonary fibrosis is the most commonly used model of idiopathic pulmonary fibrosis for studying disease pathogenesis and testing of novel pharmaceutical compounds [5]. Nevertheless, the mechanisms of BLM-induced pulmonary fibrosis are not completely understood.

Endoplasmic reticulum (ER) is an important organelle required for normal cellular function. In the ER, nascent proteins are folded with the assistance of ER chaperones. If nascent proteins in the ER are excessive compared with the reserve of ER chaperones, ER stress occurs. Accumulation of unfolded and misfolded proteins aggregated in the ER lumen causes the activation of a signal response termed the unfolded protein response (UPR) [6]. The UPR signaling is mediated by three transmembrane ER proteins: inositol requiring ER-to-nucleus signal kinase (IRE)1, activating transcription factor (ATF)6 and double-stranded RNA-activated kinase (PKR)-like ER kinase (PERK) [7], [8]. Active IRE1 cleaves *x-box binding protein- 1* (*XBP-1*) mRNA in a site-specific manner to remove an intron, promoting its unconventional splicing to generate an active transcription factor [9]. Active ATF6 is a transcription factor that promotes the expression of genes that include glucose regulated protein 78 (GRP78), ATP-dependent protein chaperone localized within the ER lumen where it promotes protein folding [10]. PERK is an ER stress-sensitive eukaryotic initiation factor (eIF) 2α kinase. Following ER stress, PERK phosphorylates and activates eIF2α, thus attenuating capdependent translation [11]. An earlier report observed an increased expression of the processed p50 ATF6 and spliced XBP-1, a downstream molecule of IRE1 pathway, in the lungs of IPF patients [12]. Two recent studies have demonstrated that pulmonary ER stress is involved in the pathogenesis of BLM-induced lung fibrosis [13], [14].

Melatonin is the major secretory product of the pineal gland. As a potent antioxidant, melatonin and its metabolites directly scavenge a variety of free radicals [12], [13]. In addition, melatonin reduces free radical levels via stimulating the activities of antioxidative enzymes [15]–[20]. Melatonin has an anti-inflammatory effect. Several studies have demonstrated that melatonin alleviates lipopolysaccharide (LPS)-evoked inflammatory cytokines/chemokines [21]–[26]. According to a recent report, melatonin attenuates LPS-induced acute lung inflammation in sleep-deprived mice [27]. Moreover, two studies found that melatonin protected against BLM-induced pulmonary fibrosis [28]–[30]. Nevertheless, the exact mechanism remains obscure.

In the present study, we investigated the effects of melatonin on BLM-induced pulmonary ER stress and the UPR in mice. We also investigated whether melatonin alleviates the EMT in the process of BLM-induced lung fibrosis. We show that melatonin protects against BLM-induced lung fibrosis in mice. We demonstrate for the first time that melatonin inhibits pulmonary ER stress and EMT during BLM-induced lung fibrosis.

## Materials and Methods

### Chemicals and reagents

BLM and melatonin were from Sigma Chemical Co. (St. Louis, MO). XBP-1, α-SMA, GAPDH and phosphor-JNK antibodies were from Santa Cruz Biotechnologies (Santa Cruz, CA). GRP78, phosphor-IRE1α, ATF6, and phosphor-eIF2α antibodies were from Cell Signaling Technology (Beverley, MA). Chemiluminescence (ECL) detection kit was from Pierce Biotechnology (Rockford, IL). All other reagents were purchased from Sigma Chemical Co. (St. Louis, MO) if not otherwise stated.

### Animals

Adult male CD-1 mice (8 week-old, 28–32 g) were purchased from Beijing Vital River whose foundation colonies were all introduced from Charles River Laboratories, Inc. The animals were allowed free access to food and water at all times and maintained on a 12-h light/dark cycle in a controlled temperature (20–25°C) and humidity (50±5%) environment. This study was approved by the Association of Laboratory AnimalSciences and the Center for Laboratory Animal Sciences at Anhui Medical University (Permit Number: 12-0046). All procedures on animals followed the guidelines for humane treatment set by the Association of Laboratory Animal Sciences and the Center for Laboratory Animal Sciences at Anhui Medical University.

### Animal model and experimental treatments

For the induction of pulmonary fibrosis, mice were intratracheally injected with BLM (5.0 mg/kg body weight in 50 µL phosphate buffered saline). To investigate the effects of melatonin on BLM-induced pulmonary fibrosis, mice were intraperitoneally (i.p.) injected with melatonin (5 mg/kg) daily, beginning at 30 min before BLM. Melatonin was dissolved in 10% ethanol and further diluted in saline (0.09% NaCl w/v) to give a final concentration of 1% ethanol. Thus, control mice received an i.p. injection of 1% ethanol daily as a control for the melatonin injections. All mice were euthanized by exsanguination during pentobarbital anesthesia (75 mg/kg,i.p.) 21 days after BLM injection. Lungs were weighed and relative lung weights were calculated. Lung fibrosis was assessed by pulmonary hydroxyproline content as well as lung histology. Some lung samples were collected and kept at –80°C for subsequent immunoblots.

### Hydroxyproline assay

Pulmonary collagen content was determined by the measurement of hydroxyproline content. In brief, lung lobes were homogenized in 1 mL of phosphate buffered saline (PBS, pH = 7.4) and then hydrolyzed in 1 mL of 6 N hydrochloric acid for 16 hours at 110°C, and neutralized to pH 7.0 with NaOH. Chloramines T reagent (1 mL of 0.5 mol/L) was then added and the samples were left at room temperature for 20 minutes. Then 20% p-Dimethylaminobenzaldehyde solution (dissolved in 3.15 N perchloric acid) was added to each sample, and the mixture was incubated at 60°C for 15 minutes. Absorbance was measured at 550 nm. Pulmonary hydroxyproline content was expressed as mg/lung.

### Pulmonary histology

Lung tissues were fixed in 4% formalin and embedded in paraffin according to the standard procedure. Paraffin-embedded lung tissues were serially sectioned. At least five consecutive longitudinal sections were stained with hematoxylin and eosin (H&E) and scored for the extent of pathology on a scale of 0 to 5, where 0 was defined as no lung abnormality, and 1, 2, 3, 4, and 5 were defined as the presence of inflammation involving 10%, 10–30%, 30–50%, 50–80%, or >80% of the lungs, respectively. Lung fibrosis was evaluated by Sirius red staining for collagen accumulation. The percentages of collagen deposition areas were quantified using NIH ImageJ software (http://rsb.info.nih.gov/ij/).

### Immunoblots

Total pulmonary lysate was prepared by homogenizing 50 mg lung tissue in 300 µl lysis buffer (50 mM Tris-HCl, pH 7.4, 150 mM NaCl, 1 mM EDTA, 1% Triton X-100, 1% sodium deoxycholate, 0.1% sodium dodecylsylphate, 1 mM phenylmethylsulfonyl fluoride) supplemented with a cocktail of protease inhibitors (Roche). For nuclear protein extraction, total pulmonary lysate was suspended in hypotonic buffer and then kept on ice for 15 min. The suspension was then mixed with detergent and centrifuged for 30 s at 14,000×g. The nuclear pellet obtained was resuspended in complete lysis buffer in the presence of the protease inhibitor cocktail, incubated for 30 min on ice, and centrifuged for 10 min at 14,000 × g. Protein concentrations were determined with the bicinchoninic acid (BCA) protein assay reagents (Pierce, Rockford, IL) according to manufacturer's instructions. For immunoblots, same amount of protein (30–60 µg) was separated electrophoretically by SDS-PAGE and transferred to a polyvinylidene fluoride membrane. The membranes were incubated for 2 h with the following antibodies: XBP-1, α-SMA, GAPDH, phosphor-JNK, GRP78, phosphor-IRE1α, ATF6, and phosphor-eIF2α. For total proteins, GAPDH was used as a loading control. For nuclear protein, lamin A/C was used as a loading control. After washes in DPBS containing 0.05% Tween-20 four times for 10 min each, the membranes were incubated with goat anti–rabbit IgG or goat anti–mouse antibody for 2 h. The membranes were then washed for four times in DPBS containing 0.05% Tween-20 for 10 min each, followed by signal development using an ECL detection kit. The density of the specific bands was quantified using NIH ImageJ software (http://rsb.info.nih.gov/ij/).

### Immunohistochemistry

For immunohistochemistry, paraffin-embedded lung sections were deparaffinized and rehydrated in a graded ethanol series. After antigen retrieval and quenching of endogenous peroxidase, sections were incubated with α-SMA monoclonal antibodies (1∶200 dilution) at 4°C overnight. The color reaction was developed with HRP-linked polymer detection system and counterstaining with hematoxylin.

### Statistical analysis

Normally distributed data were expressed as means ± SEM. ANOVA and the Student-Newmann-Keuls post hoc test were used to determine differences among different groups. Data that were not normally distributed were assessed for significance using non-parametric tests techniques (Kruskal-Wallis test and Mann–Whitney U test). P <0.05 was considered to indicate statistical significance.

## Results

### Effects of melatonin on BLM-induced pulmonary fibrosis

**Figure 1:**
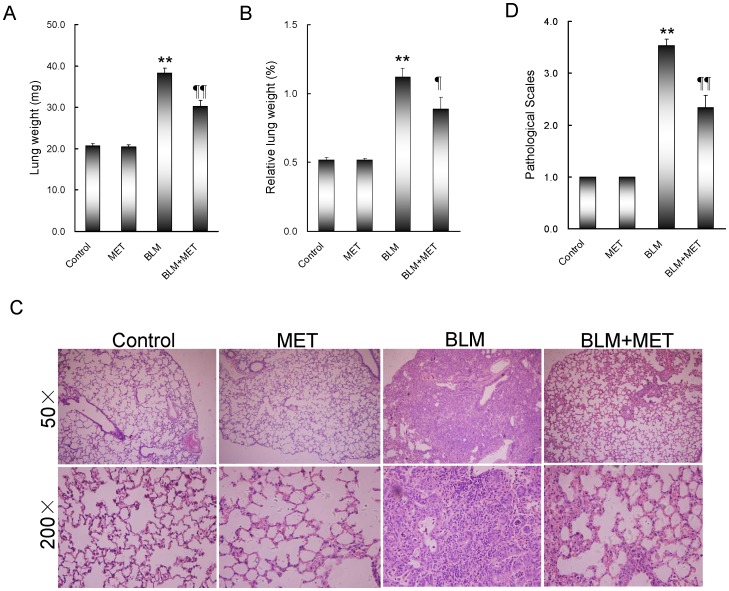
The effects of melatonin on BLM-induced pathohistological damage. All mice except controls were intratracheally injected with BLM (5.0 mg/kg). In BLM+melatonin group, mice were i.p. injected with melatonin (5 mg/kg) daily. Lungs were collected at 21 days after BLM. Lungs were weighed. (A) Absolute lung weight. (B) Relative lung weight. (C) Lung cross sections were stained with H & E. Original magnification: 50× (upper) and 200× (lower). (D) Pathohistological scores were evaluated according to pulmonary inflammation. All data were expressed as means ± SEM (n = 12). ** *P*<0.01 as compared with control group. ‡ *P*<0.05, ‡‡ *P*<0.01 as compared with BLM group.

**Figure 2:**
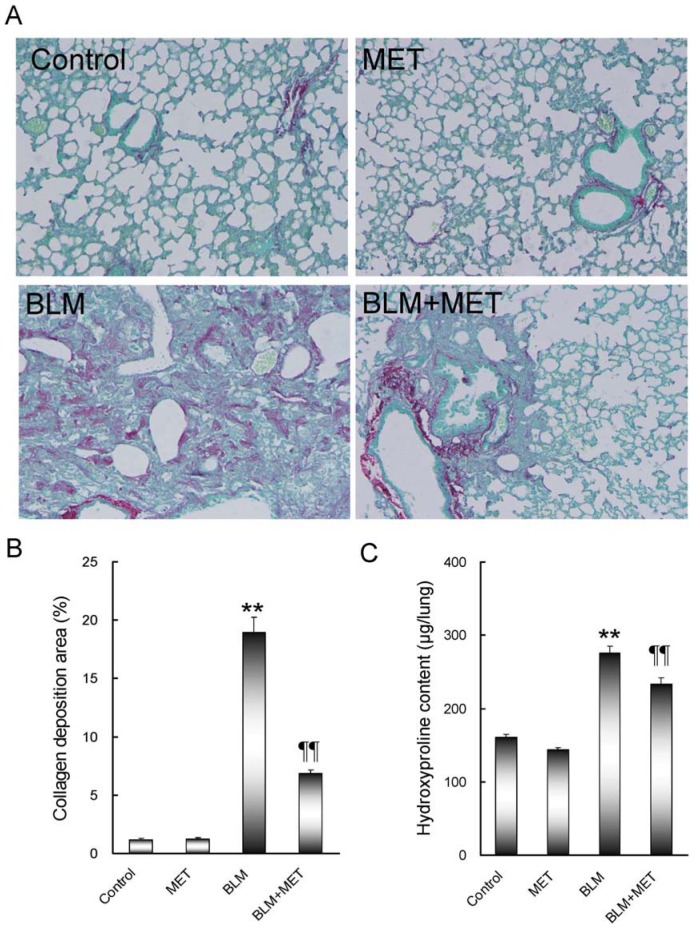
The effects of melatonin on BLM-induced pulmonary fibrosis. All mice except controls were intratracheally injected with BLM (5.0 mg/kg). In BLM+melatonin group, mice were i.p. injected with melatonin (5 mg/kg) daily. Lungs were collected at 21 days after BLM. (A) Lung fibrosis was evaluated by Sirius red staining. Original magnification: 100×. (B) Collagen deposition areas were quantified. (C) Hydroxyproline content in the lungs was analyzed. All data were expressed as means ± SEM (n = 12). ** *P*<0.01 as compared with control group. ‡‡ *P*<0.01 as compared with BLM group.

An obvious pulmonary edema was observed in BLM-treated mice. Consistent with pulmonary edema, the absolute and relative weights of the lungs were significantly increased in BLM-treated mice (Figures 1A and 1B). Of interest, melatonin significantly attenuated BLM-induced pulmonary edema. Correspondingly, melatonin significantly alleviated BLM-induced elevation of the absolute and relative lung weight (Figure 1A and 1B). Histological examination showed that melatonin significantly alleviated BLM-induced infiltration of inflammatory cells in the lungs (Figure 1C and 1D). The hallmark characteristic of BLM-induced pulmonary fibrosis is the excessive deposition of an extracellular matrix, such as collagen. As shown in Figures 2A and 2B, an obvious matrix protein deposition, as evidenced by Sirius red staining, was observed in the lungs of BLM-treated mice. As expected, melatonin significantly attenuated BLM-induced matrix protein deposition in the lungs. Moreover, melatonin significantly attenuated BLM-induced elevation of hydroxyproline content in the lungs (Figure 2C).

### Effects of melatonin on BLM-induced epithelial-mesenchymal transition

**Figure 3:**
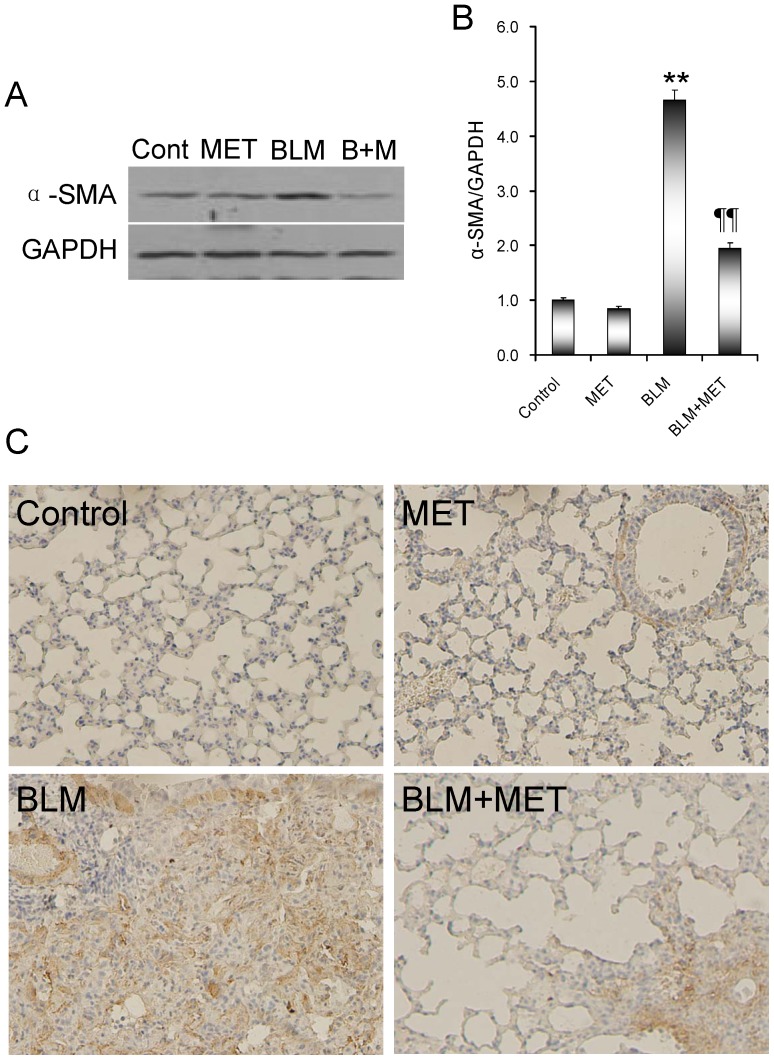
The effects of melatonin on BLM-induced pulmonary α-SMA expression. All mice except controls were intratracheally injected with BLM (5.0 mg/kg). In BLM+melatonin group, mice were i.p. injected with melatonin (5 mg/kg) daily. Lungs were collected at 21 days after BLM. (A and B) The expression of pulmonary α-SMA was detected using immunoblots. All experiments were replicated for four times. The data were expressed as means ± SEM (n = 4). ** *P*<0.01 as compared with control group. ‡‡ *P*<0.01 as compared with BLM group. (C) Pulmonary α-SMA was detected by immunohistochemistry. All experiments were replicated for four times. Original magnification: 200×.

Alpha-SMA is a hallmark of myofibroblasts and is also accepted as a marker of pulmonary fibrosis. As shown in Figures 3A and 3B, α-SMA was up-regulated in the lungs of BLM-treated mice. Immunohistochemistry showed that α-SMA was expressed in the area of pulmonary fibrosis in BLM-treated mice (Figure 3C). Of interest, melatonin significantly attenuated BLM-induced up-regulation of α-SMA in the lungs (Figures 3A, 3B and 3C).

### Effects of melatonin on BLM-induced pulmonary ER stress

**Figure 4:**
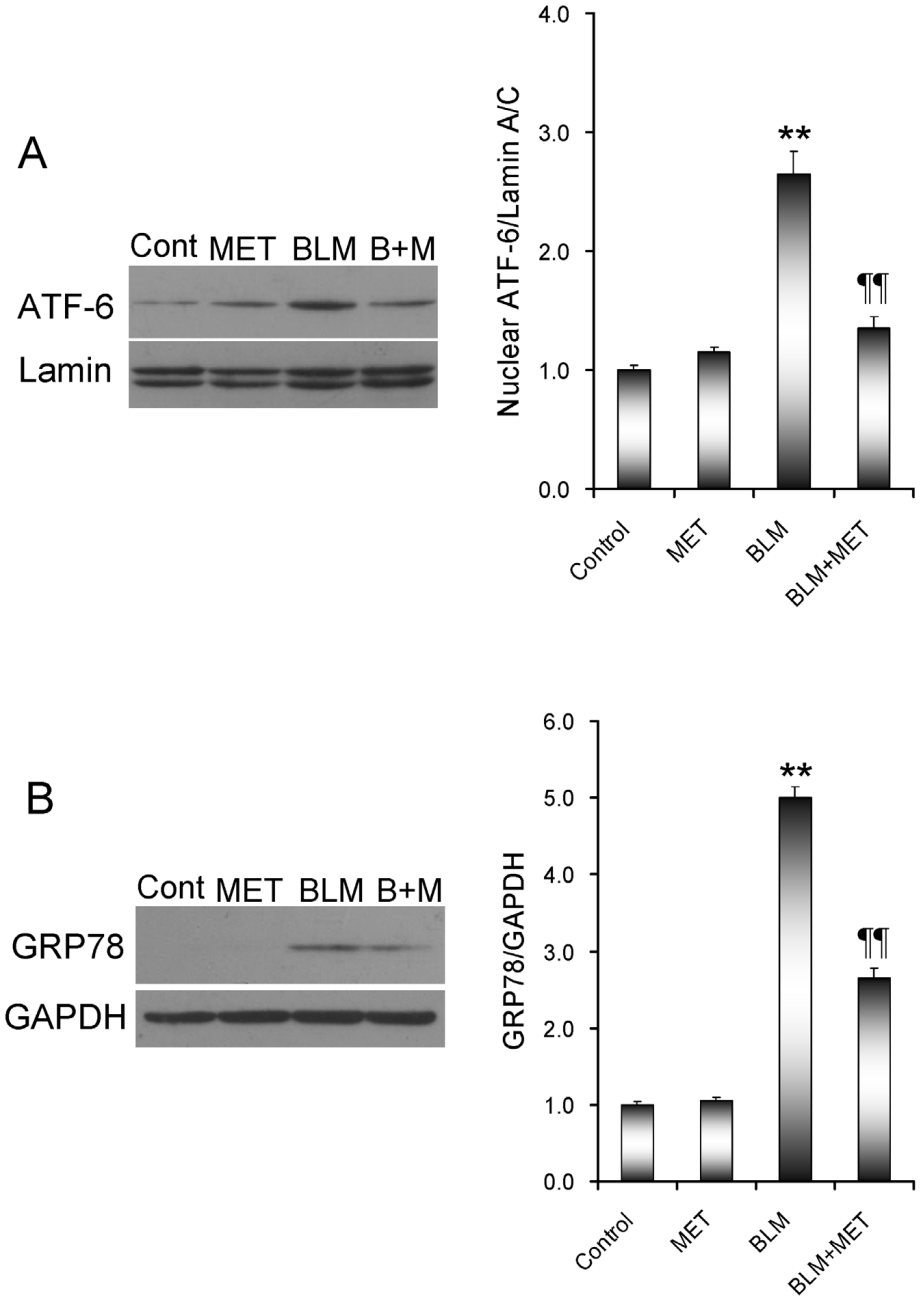
The effects of melatonin on BLM-induced activation of ATF6 pathway in the lungs. All mice except controls were intratracheally injected with BLM (5.0 mg/kg). In BLM+melatonin group, mice were i.p. injected with melatonin (5 mg/kg) daily. Lungs were collected at 21 days after BLM. (A) The cleaved ATF6 in the nuclei was detected using immunoblots. (B) GRP78 in the lungs was detected using immunoblots. All experiments were replicated for four times. The data were expressed as means ± SEM (n = 4). ** *P*<0.01 as compared with control group. ‡‡ *P*<0.01 as compared with BLM group.

**Figure 5:**
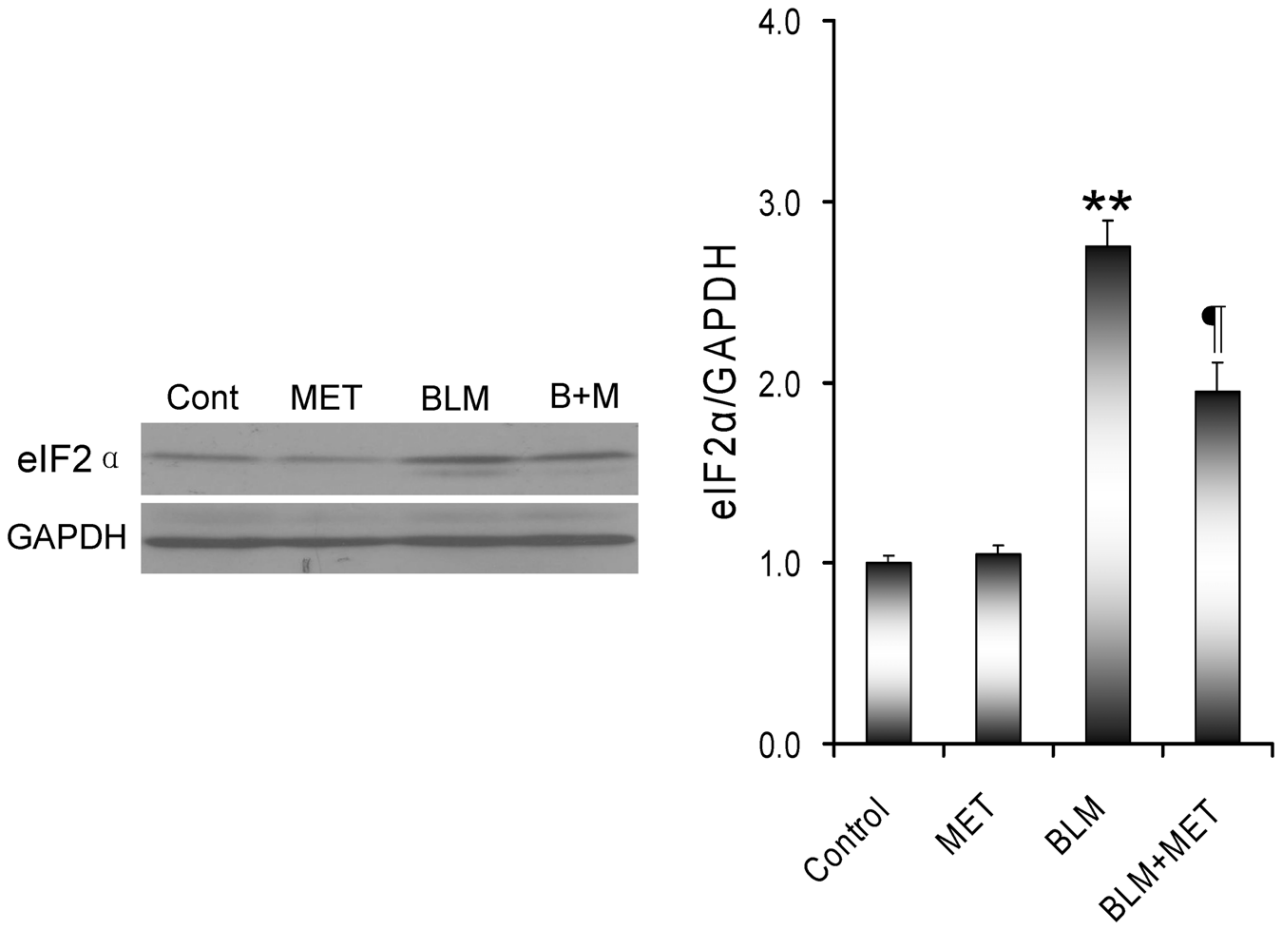
The effects of melatonin on BLM-induced eIF2α phosphorylation in the lungs. All mice except controls were intratracheally injected with BLM (5.0 mg/kg). In BLM+melatonin group, mice were i.p. injected with melatonin (5 mg/kg) daily. Lungs were collected at 21 days after BLM. Pulmonary peIF2α was detected using immunoblots. All experiments were replicated for four times. The data were expressed as means ± SEM (n = 4). ** *P*<0.01 as compared with control group. ‡ *P*<0.05 as compared with BLM group.

**Figure 6:**
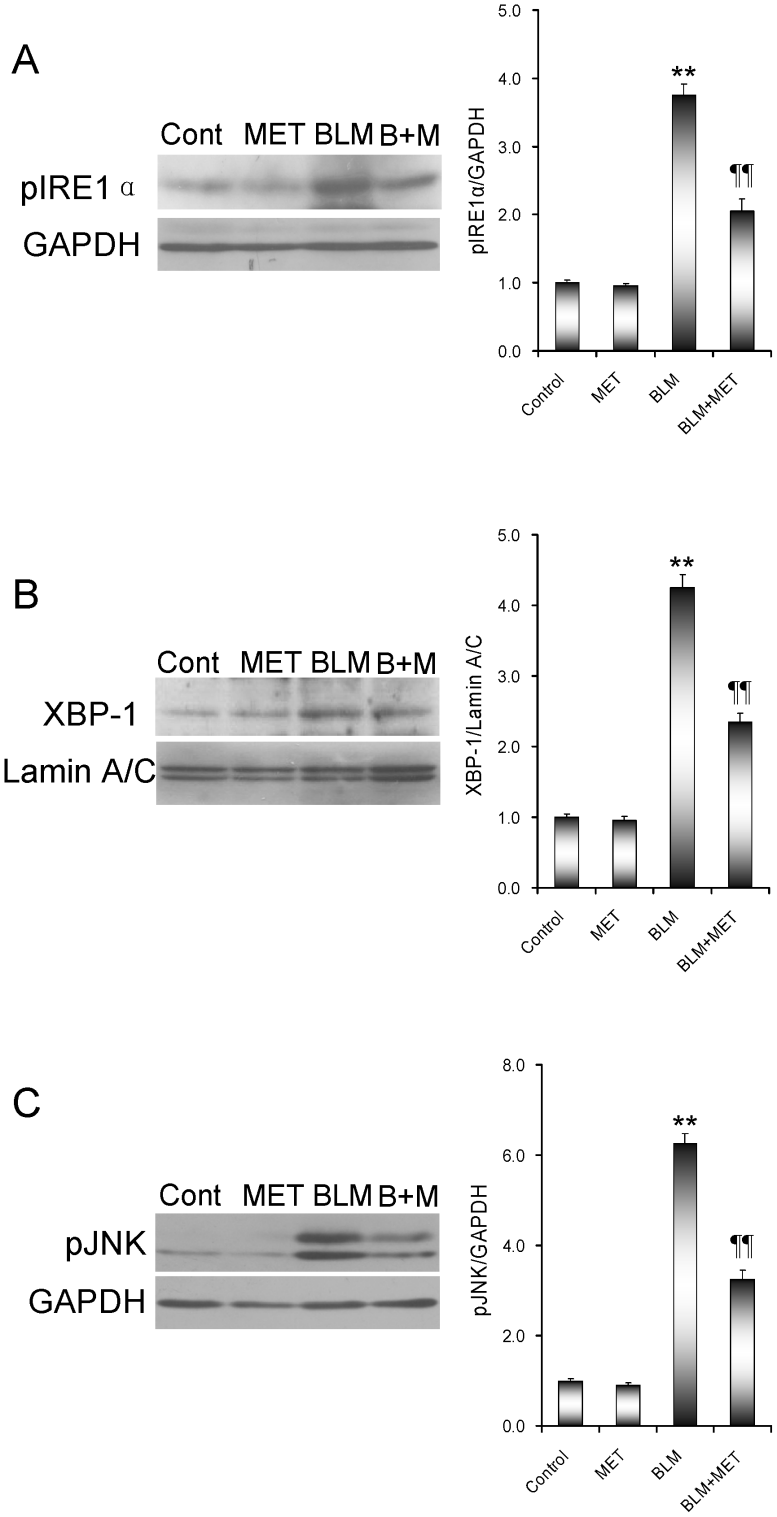
The effects of melatonin on BLM-induced activation of IRE1 pathway in the lungs. All mice except controls were intratracheally injected with BLM (5.0 mg/kg). In BLM+melatonin group, mice were i.p. injected with melatonin (5 mg/kg) daily. Lungs were collected at 21 days after BLM. (A) Pulmonary pIRE1α was detected using immunoblots. (B) Nuclear XBP-1 was detected using immunoblots. (C) Pulmonary pJNK was detected using immunoblots. All experiments were replicated for four times. The data were expressed as means ± SEM (n = 4). ** *P*<0.01 as compared with control group. ‡‡ *P*<0.01 as compared with BLM group.

The effects of melatonin on BLM-induced pulmonary ER stress were analyzed. As shown in Figure 4A, the cleaved ATF6 in the nuclei was significantly increased in the lungs of mice treated with BLM. Correspondingly, pulmonary GRP78, an ER chaperone and the target of ATF6 pathway, was up-regulated in BLM-treated mice (Figure 4B). Of interest, melatonin markedly attenuated BLM-induced elevation of the cleaved ATF6 in the lungs. Moreover, melatonin inhibited BLM-induced up-regulation of pulmonary GRP78. Eukaryotic initiation factor 2α (eIF2α) is a downstream target of the PERK pathway. As shown in Figure 5, phosphorylated eIF2α in the lung was increased in BLM-treated mice. Of interest, melatonin significantly attenuated BLM-induced eIF2α phosphorylation in the lungs. The effects of melatonin on pulmonary IRE1 pathway were then analyzed. As shown in Figure 6A, the level of phosphorylated IRE1α in the lungs was significantly increased in BLM-treated mice. Consistent with IRE1α phosphorylation, nuclear XBP-1, a downstream target of the IRE1α pathway, was elevated in the lungs of BLM-treated mice (Figure 6B). JNK, another downstream target of the IRE1 signaling, was activated in the lungs of BLM-treated mice (Figure 6C). As expected, melatonin significantly attenuated BLM-induced pulmonary IRE1α phosphorylation (Figure 6A). Moreover, melatonin significantly alleviated BLM-evoked activation of XBP-1 and JNK in the lungs (Figure 6B and 6C).

## Discussion

The present study showed an obvious matrix protein deposition, as determined by Sirius red staining, in the lungs of BLM-treated mice. Moreover, an obvious rise in hydroxyproline content was observed in the lungs when mice were injected with BLM. Two earlier reports demonstrate that melatonin alleviates BLM-induced lung fibrosis in rats [28], [30]. The present study investigated the effects of melatonin on BLM-induced lung fibrosis in mice. We showed that melatonin significantly alleviated BLM-induced matrix protein deposition in the lungs. Moreover, melatonin obviously attenuated BLM-induced elevation of pulmonary hydroxyproline content. These results suggest that melatonin protects against BLM-induced lung fibrosis in mice.

The ER is an important organelle required for normal cellular function. In the ER, nascent proteins are folded with the assistance of ER chaperones. The ER is sensitive to alterations in cellular homeostasis. When unfolded and misfolded proteins are retained in the ER lumen, ER stress occurs and the unfolded protein response (UPR) is activated [31]–[33]. Several studies demonstrate that ER stress and activation of the UPR signaling are involved in the pathogenesis of idiopathic pulmonary fibrosis [34]. According to several earlier reports, ER stress and UPR activation are observed in the alveolar epithelium in patients with idiopathic pulmonary fibrosis [12], [35]. A recent study indicates that ER stress enhances BLM-evoked fibrotic remodeling in the lungs [14]. In the present study, we showed that a single dose of BLM injection caused pulmonary ER stress and activation of the UPR signaling in the lungs. First, the cleaved ATF6 in the nuclei was activated in the lungs of BLM-treated mice. GRP78, an ER chaperone and the target of ATF6 pathway, was up-regulated in the lungs of BLM-treated mice. Next, phosphorylated eIF2α, a downstream target of the PERK signaling, was markedly increased in the lungs of BLM-treated mice. Finally, phosphorylated IRE1α in the lungs was significantly increased when mice were injected with BLM. Correspondingly, pulmonary XBP-1 and JNK, two downstream targets of the IRE1α signaling, were activated by BLM.

Melatonin is an inhibitor of ER stress. Previous report from our laboratory showed that melatonin obviously attenuated lipopolysaccharide-induced placental ER stress in mice [36]. Indeed, melatonin significantly repressed arsenite-induced elevations in activating transcription factor-4, CCAAT/enhancer binding protein homologues protein (CHOP) and GRP78 and activation of XBP-1 in rat brain [37]. According to a recent report, melatonin attenuates cadmium-induced activation of XBP-1, up-regulation of GRP78 and CHOP, and phosphorylation of eIF2α and JNK in testes [38]. Another recent report demonstrated that melatonin significantly alleviated ER stress and modulates the UPR signaling in rabbits with lethal fulminant hepatitis [39]. In the present study, we investigated the effects of melatonin on pulmonary ER stress and the UPR signaling during BLM-induced lung fibrosis. We found that melatonin alleviated BLM-induced elevation of the cleaved ATF6 in the lungs. In addition, melatonin attenuated BLM-induced up-regulation of GRP78 in the lungs. Moreover, melatonin significantly attenuated BLM-evoked pulmonary eIF2α phosphorylation. Finally, melatonin markedly alleviated BLM-induced pulmonary IRE1α phosphorylation. Correspondingly, melatonin significantly attenuated BLM-induced elevation of nuclear XBP-1 and JNK phosphorylation in the lungs. These results suggest that melatonin inhibits pulmonary ER stress and activation of the UPR signaling during BLM-induced lung fibrosis. Melatonin-mediated protection against BLM-induced lung fibrosis might be associated with its alleviation of ER stress in the lungs.

Increasing evidence demonstrates that EMT of alveolar epithelial cells to myofibroblasts plays a potential role in the pathogenesis of idiopathic pulmonary fibrosis [40], [41]. Indeed, pulmonary α-SMA, a hallmark of EMT to myofibroblasts, was up-regulated during BLM-induced pulmonary fibrosis in rodent animals [42]–[44]. According to two recent reports, pulmonary ER stress contributes to EMT in alveolar epithelial cells as a possible mechanism for fibrotic remodeling [13], [34]. In the present study, we showed that melatonin significantly alleviated BLM-induced pulmonary ER stress and activation of the UPR signaling. Correspondingly, melatonin blocked BLM-induced EMT to myofibroblasts in the lungs, as evidenced by its repression of pulmonary α-SMA. Taken together, these results suggest that melatonin protects against BLM-induced pulmonary fibrosis through its inhibition of ER stress-mediated EMT in the lungs.

In summary, the present study indicates that ER stress and activation of the UPR signaling in the lungs are involved in the pathogenesis of BLM-induced lung fibrosis. We demonstrate for the first time that melatonin inhibits BLM-induced pulmonary ER stress and ER stress-mediated EMT in the lungs. Importantly, melatonin protects against BLM-induced pulmonary fibrosis in mice. Therefore, melatonin may be useful as pharmacological agents to protect against idiopathic pulmonary fibrosis.
