# Correction: Melatonin Inhibits Endoplasmic Reticulum Stress and Epithelial-Mesenchymal Transition during Bleomycin-Induced Pulmonary Fibrosis in Mice

**DOI:** 10.1371/journal.pone.0119381

**Published:** 2015-03-25

**Authors:** The PLOS ONE Staff

The eighth author’s name is spelled incorrectly. The correct name is: Rong-Yu Liu. The correct initials in the Author Contributions are RYL.
